# Inverse association between isoflavones and prediabetes risk: evidence from NHANES 2007–2010 and 2017–2018

**DOI:** 10.3389/fnut.2023.1288416

**Published:** 2023-12-05

**Authors:** Yanjun Zhou, Shaolei Qin, Yan Zhu, Peng Xu, Ke Gu

**Affiliations:** ^1^Department of Radiotherapy and Oncology, The Affiliated Hospital of Jiangnan University, Wuxi, Jiangsu, China; ^2^Wuxi Medical School, Jiangnan University, Wuxi, Jiangsu, China; ^3^Population Health Sciences, German Centre for Neurodegenerative Diseases (DZNE), Bonn, Germany

**Keywords:** prediabetes, isoflavones, glycitein, daidzein, genistein

## Abstract

**Introduction:**

Prediabetes is a metabolic condition characterized by blood glucose levels that are higher than normal but do not meet the threshold for a diabetes diagnosis. Individuals with prediabetes are at an increased risk of developing type 2 diabetes and associated complications. However, limited epidemiological studies have investigated the association between flavonoids from plant-based diets and the risk of prediabetes, and the existing evidence from these studies is inconsistent.

**Methods:**

Therefore, we utilized data from 19,021 participants (mean age: 32.03 years) in the National Health and Nutrition Examination Survey (NHANES) conducted during 2007–2010 and 2017–2018 to investigate the potential association between dietary flavonoid intake and prediabetes risk by weighted logistic regression analysis. Furthermore, the data from 3,706 participants (mean age: 35.98 years) from NHANES 2007–2010 were used to assess the correlation between concentrations of isoflavones and their metabolites in urine and prediabetes risk by weighted logistic regression analysis.

**Results:**

Our findings revealed an inverse association between the intake of glycitein (OR: 0.88; 95% CI: 0.82–0.96; *p* = 0.003), genistein (OR: 0.98; 95% CI: 0.97–0.99; *p* = 0.004), daidzein (OR: 0.98; 95% CI: 0.96–0.99; *p* = 0.009), and total isoflavones (OR: 0.99; 95% CI: 0.98–1.00; *p* = 0.005) with the risk of prediabetes. Moreover, we observed an inverse association between the concentration of daidzein in urine (OR: 0.84; 95% CI: 0.73–0.96; *p* = 0.012) and the concentration of genistein in urine (OR:0.83; 95% CI: 0.75–0.93; *p* = 0.003) with the risk of prediabetes using weighted logistic regression.

**Conclusion:**

In conclusion, our findings suggest a potential protective effect of isoflavones against the development of prediabetes.

## 1 Introduction

Prediabetes is defined by elevated blood glucose levels that surpass the normal range but fall short of meeting the diagnostic criteria for diabetes (1). The diagnostic criteria for prediabetes remain a topic of debate and typically encompass impaired fasting glucose [American Diabetes Association criteria: 5.6–6.9 mmol/L; International Diabetes Federation (IDF) and World Health Organization criteria: 6.1–6.9 mmol/L], impaired glucose tolerance (7.8–11.0 mmol/L), and/or glycated hemoglobin (HbA1C) levels ranging from 5.7 to 6.4% (2, 3). Prediabetes has been incorporated into the ICD-10 coding system (R73.0). According to the 9th edition of the IDF Diabetes Atlas, the age-adjusted prevalence of IGT among adults aged 20–79 years worldwide was estimated at 9.1% in 2021 and 10.0% by 2,045 (2). However, IGT only captures a subset of individuals with prediabetes. To provide a more comprehensive assessment of the global burden of prediabetes, it is necessary to also determine the prevalence of IFG. An estimated 5.8% of the global adult population aged 20–79 years had IFG in 2021 and 6.5% by 2,045 (2). The prevalence of prediabetes varies across different age groups, income of countries, and urban-rural areas (2). The age-adjusted prevalence of IGT in 2021 was highest in the North America and Caribbean regions (13.1%) (2). Prediabetes has a high risk of progressing to diabetes, with estimates suggesting that 5–10% of individuals with prediabetes develop diabetes each year (4–7). Prediabetes is associated with an increased risk of complications such as diabetic retinopathy, chronic kidney disease, acute coronary syndrome, and stroke (7–11). Timely intervention through lifestyle modifications, including increased physical exercise, healthy diet, and weight management, can effectively control blood glucose levels and delay the onset of diabetes while reducing complications (12, 13). However, a paucity of epidemiological studies examining the association between prediabetes and dietary flavonoids.

Flavonoids are a class of bioactive agents, abundantly present in various plant-originated foods, including fruits, vegetables, herbs, and any other edible plant parts. Flavonoids can be divided into six subclasses, including isoflavones, anthocyanidins, flavan 3-ols, flavanones, flavones, and flavonols. Among flavonoids, isoflavones, including daidzein, genistein, and glycitein, are abundant in legumes, specifically soybeans and soy products. Although the blood glucose control effects of isoflavones have been observed in animal experiments (14), there is currently a limited amount of epidemiological research and clinical trials examining the association between isoflavones and prediabetes (15). Therefore, our study aims to fill a research gap by investigating the relationship between flavonoids, particularly isoflavones, and prediabetes risk. In this study, we excluded individuals with diabetes mellitus (DM) and stratified the population previously considered healthy based on the diagnostic criteria for prediabetes (16). The participants without DM were categorized into a normoglycemic group and a prediabetes group. Moreover, currently, most epidemiological studies and clinical trials have focused on the impact of dietary isoflavones on the incidence of type 2 diabetes (T2D). However, these studies have yielded inconsistent conclusions (16–18), and there is a lack of research investigating whether the inconsistent conclusions are attributed to varying disease severity or the extent of pancreatic β cell damage. By investigating prediabetes, we intend to investigate whether individuals in the early stages of abnormal glucose metabolism correspond to the potential beneficiary population of isoflavones.

To achieve the aforementioned objectives, the association between isoflavones and the risk of prediabetes was assessed by diverse indicators, including the dietary flavonoids consumption from National Health and Nutrition Examination Survey (NHANES) and USDA Food Code Flavonoid Values Database as well as the concentrations of isoflavones and their metabolic byproducts in the urine from NHANES.

## 2 Materials and methods

### 2.1 Study population

**FIGURE 1:**
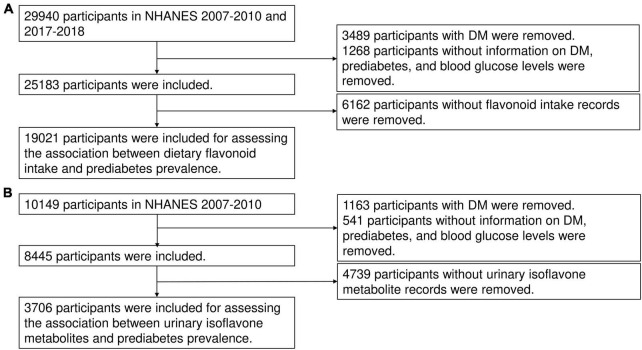
The flow chart of this study. The flow chat of inclusion and exclusion for assessing the association between dietary flavonoid intake and prediabetes risk **(A)** and the association between urinary isoflavone metabolites and prediabetes risk **(B)**.

To assess the relationship between dietary flavonoid consumption and prediabetes risk, we utilized epidemiological information and complete data on dietary flavonoid consumption of individuals aged above 18 years in the NHANES cycles from 2007 to 2010 and 2017 to 2018. NHANES is a nationally representative, cross-sectional study conducted periodically since the 1960s with the objective of evaluating the health and nutrition status of both children and adults in the United States (US). The study population is recruited through a comprehensive multistage, stratified sampling design, ensuring the representation of diverse demographic groups (19, 20). A total of 29,940 participants with available sociodemographic data were initially included in the study. Exclusions were made for individuals without information on diabetes or prediabetes history (*N* = 1,268) as well as those with DM (*N* = 3,489), resulting in a total of 25,183 participants remained. Participants with missing data on flavonoid intake (*N* = 6,162) were also excluded. Consequently, the analysis focused on a total of 19,021 participants to determine the association between dietary flavonoid intake and the risk of prediabetes (Figure 1A). Furthermore, to explore the relationship between prediabetes risk and urinary levels of isoflavones and their metabolites, data from the NHANES cycles conducted between 2007 and 2010 were utilized (*N* = 10,149). Individuals with missing data on incomplete information were excluded from this particular analysis (*N* = 6,443). Ultimately, a total of 3,706 participants were considered in assessing the association between urinary isoflavone metabolites and prediabetes risk (Figure 1B). The study was carried out in accordance with the principles delineated in the Declaration of Helsinki and obtained approval from the Institutional Review Board (IRB) or Ethics Committee of the National Center for Health Statistics, CDC (Protocol #2005-06, #2011-17, #2018-01). All adult participants provided written informed consent prior to their inclusion in the NHANES.

### 2.2 Assessment of flavonoid intakes

The values of dietary flavonoid intake were accessed from the flavonoid database, which was briefly described in our previous paper (21). The mean of each flavonoid’s 2-day intake based on the dietary recall was considered as the consumption of flavonoids. The weight “wtdr2d” was employed in weighted analysis concerning the relationship between dietary flavonoid intake and the risk of prediabetes. Besides, the primary flavonoid subclasses in Supplementary Table 1. The weights “wtsb2yr” for the 2007–2008-year cycle and “wtsa2yr” for the 2008–2010-year cycle were used in the weighted analysis on the association between urinary isoflavones and the risk of prediabetes.

### 2.3 Assessment of prediabetes

The diagnostic criteria for prediabetes encompass a clinical diagnosis by a medical professional, specific HbA1c ranging from 5.7 to 6.5%, fasting glucose levels between 5.6 to 7.0 mmol/l, and 2-h blood glucose levels during the oral glucose tolerance test (OGTT2) ranging from 7.8 to 11.0 mmol/l (1, 3). The established diagnostic criteria for DM encompass a medical practitioner’s clinical diagnosis of diabetes, HbA1c levels going beyond 6.5%, fasting glucose levels equal to or greater than 7.0 mmol/l, random blood glucose levels equal to or greater than 11.1 mmol/l, OGTT2 equal to or greater than 11.1 mmol/l (1, 3), and the utilization of antidiabetic agents or insulin therapy.

### 2.4 Assessment of covariates

Demographic information was acquired through the administration of questionnaires during the initial interview at the participants’ home. The data included variables such as age, sex, ethnic background, academic level, poverty income ratio (PIR), partner status, smoking habits, alcohol consumption, total duration of physical activity (PA) per week, and total metabolic equivalent (MET) of PA per week. The academic level was classified into three categories: less than 9 years, 9 to 12 years, and greater than 12 years. Partner status was categorized as either partnered or unpartnered. Body mass index (BMI) was computed as kg/m^2^. Smoking habits were stratified into three groups: (1) never smoked, defined as having cumulative cigarette consumption below 100 throughout one’s lifetime; (2) former smoker, characterized by cumulative cigarette consumption exceeding 100 throughout one’s lifetime; and (3) current smoker, defined as an individual who has consumed exceeding 100 cigarettes throughout one’s lifetime and currently engages in smoking either intermittently or daily. Consumption of alcohol was classified as in Rattan et al. (22). The healthy eating index (HEI) for the 2015 Edition was calculated by aggregating the dietary intake data collected over two consecutive days (23). The dietary inflammatory index (DII) was derived by employing the following calculation method: (24).

Information regarding disease history was collected through in-home interviews and laboratory measurements. Hyperlipidemia was defined upon meeting any of the following criteria: self-report answers to the question: “Have you ever been told by a doctor that you have hyperlipidemia?” Those who answer “Yes” were considered to have hyperlipidemia, triglycerides equal to or exceeding 150 mg/dL, low-density lipoprotein cholesterol equal to or surpassing 130 mg/dL, high-density lipoprotein cholesterol lower than 140 ng/dL, or use of antihyperlipidemic medications. A prior occurrence of stroke is regarded as a medical history of stroke. A previous diagnosis of any form of cancer is acknowledged as a cancer medical history. The average blood pressure was determined in accordance with (25), and hypertension was defined when any of the following criteria were satisfied: previous diagnosis of hypertension, use of antihypertensive medications or systolic pressure equal to or greater than 140 mmHg or diastolic pressure equal to or greater than 90 mmHg on three separate occasions. The criteria utilized to determine a history of cardiovascular disease (CVD) involve the presence of a medically confirmed diagnosis of either coronary heart disease, congestive heart failure, myocardial infarction, cerebrovascular accident, or angina.

### 2.5 Statistical analysis

R software (version 4.1.3) was utilized for all statistical analyses. Data cleaning and weighted statistical analyses were conducted by the packages “NHANESR” and “survey” in R. The missing covariate values were imputed by the “mice” package in R. SDMVPSU and SDMVSTRA were utilized to calculate nationally representative estimates in NHANES. In the baseline analysis, descriptive statistics for continuous variables were presented as mean ± standard deviation, median, and percentile range, while categorical variables were displayed as percentages. Categorical variables were compared using the chi-square test, while continuous variables were compared using the Wilcoxon test. Subsequently, age-adjusted and sex adjusted mean dietary flavonoid intakes were compared across the ethnic groups by using linear regression. Moreover, Logistic regression analysis was employed to investigate the potential relationship between total flavonoid intake and prediabetes risk. Model 1 was crude model with no adjustment. Model 2 was adjusted with the adjustment for age, sex, race, BMI, daily energy intake, total time of PA, smoking habits, alcohol consumption, hyperlipidemia, and hypertension. Model 3 was adjusted with the adjustment for age, sex, race, BMI, daily energy intake, total time of PA, smoking habits, alcohol consumption, hyperlipidemia, hypertension and survey year cycle. The inclusion of variables in the model was determined based on the literature (26). The concentration of isoflavone metabolites in urine was not normally distributed. Therefore, a logarithmic transformation (base 10) was applied to achieve normality. Subsequently, the weighted logistic regression analysis was carried out treating the log10-transformed concentration of isoflavone metabolites in urine as a continuous variable. To confirm the association, the concentrations of urinary metabolites were categorized into quartiles (Q1: 0–25%, Q2: 25–50%, Q3: 50–75%, Q4: 75–100%) for logistic regression analysis. Q1 was used as the reference category, and comparisons were made between Q2, Q3, and Q4 with Q1. The *p*-values for trend were determined to assess the statistical significance of a trend across ordered quartiles, where the median values of each quartile are utilized. Model 1 was the crude model. Model 2 was adjusted for age, sex, race, BMI, and creatinine. Model 3 was adjusted for age, sex, race, BMI, daily energy intake (kcal/day), total of time of PA, smoking habits, alcohol consumption, hyperlipidemia history, and hypertension history and urinary creatinine. Weighted logistic regression for subgroup analyses was fully adjusted with the exception of the stratification factors including age, sex, race, smoking habits, alcohol consumption, PIR, BMI, total time of PA, daily energy intake, hyperlipidemia, CVD, stroke, cancer, and hypertension. Weighted logistic regression was conducted to estimate odds ratios (OR) along with their corresponding 95% confidence intervals (CIs). A significance level of *p* < 0.05 was selected as the threshold for statistical significance.

## 3 Results

### 3.1 Baseline characteristics

A total of 19,021 subjects with complete data regarding flavonoid consumption and without DM were included in our study, which can represent 269,831,563 non-hospitalized US population. A total of 4,672 (24.56%) participants were diagnosed with prediabetic status. The features of the study cohort were summarized in Table 1. Compared to those with normal blood glucose, participants with prediabetes were older (49.79, *p* < 0.0001); more frequently males (51.04%, *p* = 0.002); lower proportion of individuals with education exceeding 12 years (*p* < 0.001); richer (with higher PIR, 2.97, *p* = 0.02); more frequently with partner (61.91%, *p* < 0.0001); less non-smoker (55.47%, *p* < 0.0001); less current alcohol user (78.88%, *p* < 0.0001); with higher BMI (29.98, *p* < 0.0001), with lower DII (1.51, *p* = 0.01); with higher daily energy intake (4226.69, *p* < 0.0001) (Table 1). In addition, the participants with prediabetes had a higher risk of hyperlipidemia (77.27%, *p* < 0.0001), CVD (9.73%, *p* < 0.0001), stroke (3.09%, *p* = 0.02), cancer (11.04%, *p* < 0.0001), and hypertension (43.44%, *p* < 0.0001) (Table 1). The majority of those with prediabetes showed low intake of daidzein, genistein, glycitein, and total isoflavones (Table 1). Prediabetic participants had higher intakes of catechin, epigallocatechin, epicatechin, epicatechin 3-gallate, epigallo-catechin 3-gallate, theaflavin, thearubigins, luteolin, isorhamnetin, kaempferol, myricetin, quercetin, theaflavin-3,3′-digallate, theaflavin-3′-gallate, theaflavin 3-gallate, gallocatechin, subtotal catechins, total flavan 3-ols, total flavones, total flavonols, and total flavonoids (Table 1).

**TABLE 1:** Characteristics of participants by prediabetes status, NHANES 2007–2010, 2017–2018.

Variables	Normal blood glucose (*N* = 14,349)	Prediabetes (*N* = 4,672)	*p*-value
**Baseline sociodemographic, lifestyle, and health-related variables**			
Age	30.45 (0.35)	49.79 (0.44)	<0.0001
Sex			0.002
Female	7,521 (53.21%)	2,246 (48.96%)	
Male	6,828 (46.79%)	2,426 (51.04%)	
Race			0.05
Non-Hispanic black participants	2,953 (11.54%)	1,000 (12.29%)	
Mexican American	2,840 (10.83%)	770 (9.05%)	
Other Races	1,261 (7.41%)	403 (8.19%)	
Non-Hispanic white participants	7,295 (70.23%)	2,499 (70.46%)	
Education			<0.001
<9 years	928 (3.94%)	481 (5.32%)	
9–12 years	5,637 (34.73%)	1,847 (38.31%)	
>12 years	7,784 (61.33%)	2,344 (56.37%)	
Partner status			<0.0001
Without partner	8,345 (51.19%)	1,959 (38.09%)	
With partner	6,004 (48.81%)	2,713 (61.91%)	
Smoking status			<0.0001
Never	9,773 (65.66%)	2,542 (55.47%)	
Former	2,071 (16.76%)	1,197 (25.39%)	
Current	2,505 (17.57%)	933 (19.14%)	
Alcohol use			<0.0001
Never	1,730 (9.95%)	586 (10.17%)	
Former	1,260 (7.61%)	607 (10.95%)	
Current	11,359 (82.44%)	3,479 (78.88%)	
PIR	2.85 (0.05)	2.97 (0.06)	0.02
BMI (kg/m^2^)	24.91 (0.11)	29.98 (0.18)	<0.0001
HEI score 2015	52.05 (0.34)	52.44 (0.47)	0.36
DII	1.71 (0.04)	1.51 (0.07)	0.01
Total time of PA	1255.86 (23.46)	1324.32 (46.09)	0.1
Total MET of PA	5252.39 (144.98)	5129.30 (211.05)	0.54
Daily energy intake (kcal)	4025.77 (23.26)	4226.69 (36.86)	<0.0001
**Disease history at interview**			
Hyperlipidemia			<0.0001
No	6,250 (49.42%)	1,163 (22.73%)	
Yes	5,692 (50.58%)	3,509 (77.27%)	
CVD			<0.0001
No	6,530 (95.82%)	3,680 (90.27%)	
Yes	426 (4.18%)	526 (9.73%)	
Stroke			0.02
No	6,769 (98.03%)	4,016 (96.91%)	
Yes	181 (1.97%)	184 (3.09%)	
Cancer			<0.0001
No	6,384 (92.63%)	3,733 (88.96%)	
Yes	567 (7.37%)	470 (11.04%)	
Hypertension			<0.0001
No	12,240 (84.04%)	2,583 (56.56%)	
Yes	2,109 (15.96%)	2,089 (43.44%)	
**Intake of flavonoids (mg/day)**			
Daidzein	0.76 (0.06)	0.56 (0.07)	0.02
Genistein	1.08 (0.08)	0.76 (0.08)	0.01
Glycitein	0.15 (0.01)	0.11 (0.01)	0.01
Cyanidin	2.28 (0.12)	2.89 (0.40)	0.13
Petunidin	1.04 (0.09)	1.10 (0.11)	0.6
Delphinidin	1.49 (0.14)	1.54 (0.15)	0.76
Malvidin	4.32 (0.28)	4.99 (0.48)	0.19
Pelargonidin	1.81 (0.17)	1.51 (0.13)	0.06
Peonidin	1.79 (0.16)	1.77 (0.19)	0.91
Catechin	6.74 (0.16)	7.84 (0.22)	<0.0001
Epigallocatechin	11.63 (0.55)	17.36 (1.05)	<0.0001
Epicatechin	9.30 (0.22)	10.49 (0.28)	<0.001
Epicatechin 3-gallate	7.53 (0.37)	11.13 (0.69)	<0.0001
Epigallocatechin 3-gallate	19.97 (0.97)	28.95 (1.93)	<0.0001
Theaflavin	1.12 (0.07)	1.72 (0.13)	<0.0001
Thearubigins	64.53 (3.62)	98.77 (6.89)	<0.0001
Eriodictyol	0.16 (0.01)	0.18 (0.02)	0.36
Hesperetin	9.06 (0.29)	9.36 (0.47)	0.56
Naringenin	3.14 (0.14)	3.35 (0.19)	0.23
Apigenin	0.17 (0.02)	0.26 (0.05)	0.1
Luteolin	0.58 (0.02)	0.70 (0.03)	0.001
Isorhamnetin	0.68 (0.02)	0.85 (0.03)	<0.0001
Kaempferol	3.61 (0.09)	4.60 (0.14)	<0.0001
Myricetin	1.13 (0.03)	1.65 (0.07)	<0.0001
Quercetin	9.38 (0.19)	11.59 (0.25)	<0.0001
Theaflavin-3,3′-digallate	1.24 (0.07)	1.90 (0.15)	<0.0001
Theaflavin-3′-gallate	1.05 (0.06)	1.61 (0.12)	<0.0001
Theaflavin-3-gallate	0.89 (0.05)	1.36 (0.10)	<0.0001
Gallocatechin	1.21 (0.06)	1.77 (0.12)	<0.0001
Subtotal Catechins	56.38 (2.23)	77.54 (4.06)	<0.0001
Total Isoflavones	1.99 (0.15)	1.42 (0.16)	0.01
Total Anthocyanidins	12.72 (0.59)	13.80 (1.08)	0.27
Total Flavan 3-ols	125.22 (5.89)	182.92 (10.64)	<0.0001
Total Flavanones	12.35 (0.40)	12.88 (0.61)	0.39
Total Flavones	0.75 (0.03)	0.96 (0.07)	0.01
Total Flavonols	14.79 (0.31)	18.70 (0.42)	<0.0001
Total Sum of all 29 flavonoids	167.82 (6.38)	230.68 (10.65)	<0.0001

The continuous variables were demonstrated as weighted mean (weighted standard error), including age, PIR, score, HEI score, DII, BMI, total time of PA, and total MET of PA, daily energy intake, and the daily intake of flavonoids. The categorical variables were demonstrated as sample number (weighted percentage), including sex, ethnicity, education, partner status, smoking status, alcohol consumption, and disease history. PIR, poverty income ratio; HEI, healthy eating index; DII, dietary inflammatory index; BMI, body mass index; PA, physical activity; MET, metabolic equivalent.

Since there was a 7-year gap in the available data, the lifestyle might change. We compared the baseline variables by survey year cycle (Supplementary Table 2). In terms of educational attainment, the proportion of individuals with more than 12 years of education was highest in the 2017–2018 survey year (64.40%), showing significant differences when compared to other survey years (*p* = 0.003 for 2007 vs. 2017, *p* = 0.04 for 2009 vs. 2017). The prevalence of current smokers decreased significantly in the 2017–2018 survey year compared to the 2007–2009 survey year cycle (*p* < 0.001 for 2007 vs. 2017). Regarding alcohol consumption, the proportion of current alcohol consumers was highest in the 2017–2018 survey year (90.15%), displaying significant differences compared to other years (*p* < 0.0001 for 2007 vs. 2017, *p* < 0.0001 for 2009 vs. 2017). Participants in the 2017–2018 survey year exhibited the highest BMI compared to other survey year cycles (*p* < 0.001 for 2007 vs. 2017, *p* < 0.001 for 2009 vs. 2017). Total time and MET of PA were the lowest in the 2009–2010 survey year cycle among the three survey years (*p* < 0.0001). The risk of hyperlipidemia decreased in the 2017–2018 survey year cycle compared to the previous survey year cycle (*p* = 0.01). The intake of daidzein and total isoflavones in 2007–2008 were lower than in 2017–2018 (*p* = 0.01, *p* = 0.01, respectively). The intake of genistein, peonidin, petunidin, delphinidin, isorhamnetin, and total anthocyanidins in 2007–2009 were the lowest among three survey year cycles. The intake of eriodictyol, hesperetin, and total flavanones in 2017–2018 was the lowest among three survey-year cycles. The intake of glycitein increased by the survey-year cycle. The intake of quercetin decreased in the 2017–2018 than in 2009–2010.

### 3.2 Differences in flavonoid intake across different age groups, sexes, and races

Regarding flavonoid intake based on subclasses, we observed variations among different demographic groups, as summarized in Table 2. Non-Hispanic black participants had significantly lower intakes of catechins, anthocyanidins, flavan-3-ols, flavonols, flavones, and total flavonoids compared to non-Hispanic white participants. However, they had higher flavanone intake than non-Hispanic white participants. Among men older than 50 years, there were no significant differences in isoflavone intake between non-Hispanic black and non-Hispanic white participants. Mexican Americans showed decreased intakes of catechins, anthocyanidins, flavan-3-ols, flavonols, and total flavonoids when compared to non-Hispanic white participants. Conversely, Mexican Americans had higher flavanone intake than non-Hispanic white participants. No substantial disparities were observed in flavone and isoflavone intake in Mexican American women younger than 50 years compared to non-Hispanic white participants. Similarly, there were no notable variations in isoflavone intake among Mexican American men younger than 50 years compared to non-Hispanic white participants. However, isoflavone intake among Mexican American men older than 50 years was lower than that of non-Hispanic white participants. Women of other races younger than 50 years had higher flavanone intake compared to non-Hispanic white participants. Women over 50 years of age from other races had significantly higher subtotal catechin intake than non-Hispanic white participants. Additionally, men of other races younger than 50 years had higher flavone intake compared to non-Hispanic white participants.

**TABLE 2:** Mean flavonoid intake values, by age, sex, and race group.

	Participants (n)	Subtotal Catechins	Total Isoflavones	Total Anthocyanidins	Total Flavan 3-ols	Total Flavanones	Total Flavones	Total Flavonols	Total flavonoids
**Non-Hispanic white participants**									
Female < 50	3,374	57.97 (49.36, 66.57)	1.56 (1.10, 2.01)	13.88 (11.98, 15.79)	131.21 (112.29, 150.14)	8.98 (7.98, 9.99)	0.68 (0.61, 0.75)	13.82 (12.85, 14.78)	170.14 (149.78, 190.49)
Female > = 50	1,650	84.10 (75.57, 92.63)	2.31 (1.65, 2.97)	21.07 (18.01, 24.13)	206.22 (173.29, 239.16)	12.34 (10.86, 13.81)	1.05 (0.79, 1.31)	19.46 (18.05, 20.88)	262.45 (227.90, 297.00)
Male < 50	3,247	58.89 (49.98, 67.80)	2.00 (1.56, 2.43)	10.51 (8.58, 12.43)	131.71 (111.42, 151.99)	11.65 (10.20, 13.11)	0.78 (0.64, 0.92)	15.83 (14.71, 16.95)	172.47 (150.91, 194.04)
Male > = 50	1,523	84.23 (72.10, 96.35)	2.05 (1.09, 3.02)	18.10 (14.73, 21.46)	204.61 (172.43, 236.78)	15.42 (13.47, 17.38)	1.10 (0.94, 1.26)	21.48 (20.10, 22.86)	262.76 (229.19, 296.33)
**Non-Hispanic black participants**									
Female < 50	1,557	38.06 (32.51, 43.61)***	0.89 (0.47, 1.30)*	7.26 (6.21, 8.32)***	81.60 (67.47, 95.72)***	13.62 (11.64, 15.60)***	0.51 (0.45, 0.57)***	10.91 (10.25, 11.57)***	114.79 (100.16, 129.42)***
Female > = 50	478	54.82 (42.59, 67.04)***	1.41 (0.50, 2.33)***	10.01 (6.42, 13.59)***	119.74 (100.80, 138.67)***	14.23 (11.02, 17.44)**	0.62 (0.54, 0.69)***	13.43 (12.25, 14.61)***	159.43 (137.65, 181.22)***
Male < 50	1,419	40.24 (34.89, 45.58)***	1.80 (0.89, 2.71)*	7.13 (5.89, 8.36)***	87.86 (74.93, 100.80)***	15.30 (13.35, 17.25)***	0.44 (0.40, 0.48)***	12.39 (11.39, 13.40)***	124.93 (110.70, 139.16)***
Male > = 50	499	60.42 (47.43, 73.41)***	1.12 (0.55, 1.70)	8.15 (5.91, 10.38)***	152.48 (115.11, 189.85)***	15.90 (12.66, 19.14)**	0.67 (0.55, 0.78)***	18.09 (16.38, 19.80)***	196.41 (158.14, 234.67)***
**Mexican American**									
Female < 50	1,564	31.64 (25.16, 38.11)***	2.11 (0.03, 4.20)	7.33 (6.05, 8.61)***	62.66 (47.86, 77.47)***	15.63 (13.40, 17.85)***	0.67 (0.58, 0.77)	10.95 (10.02, 11.88)***	99.36 (83.56, 115.16)***
Female > = 50	306	37.32 (28.37, 46.28)***	1.01 (0.45, 1.58)	12.64 (4.18, 21.11)***	71.71 (52.40, 91.01)***	16.59 (13.69, 19.50)***	0.93 (0.76, 1.09)	13.33 (11.29, 15.36)***	116.21 (93.55, 138.87)***
Male < 50	1,481	37.05 (31.57, 42.53)***	1.39 (0.45, 2.33)	6.42 (4.78, 8.06)***	61.72 (50.16, 73.28)***	16.89 (15.07, 18.70)***	0.74 (0.66, 0.81)	12.76 (11.93, 13.59)***	99.92 (86.78, 113.05)***
Male > = 50	259	46.22 (22.46, 69.98)***	0.74 (0.19, 1.28)*	10.70 (3.59, 17.81)***	101.46 (38.01, 164.90)***	18.84 (12.78, 24.90)***	1.31 (0.50, 2.13)	17.29 (14.09, 20.48)***	150.34 (82.57, 218.10)***
**Other races**									
Female < 50	671	54.10 (41.77, 66.42)	2.38 (1.18, 3.58)	11.81 (9.27, 14.35)	119.00 (86.60, 151.40)	12.34 (9.66, 15.03)*	0.84 (0.70, 0.98)	15.28 (13.47, 17.08)	161.65 (127.75, 195.56)
Female > = 50	167	173.44 (108.31, 238.56)*	3.21 (1.84, 4.58)	14.94 (9.36, 20.51)	349.52 (246.81, 452.23)	14.86 (9.43, 20.29)	1.26 (0.83, 1.70)	24.31 (19.06, 29.57)	408.11 (298.28, 517.93)
Male < 50	681	68.90 (36.37, 101.43)	2.00 (1.34, 2.67)	12.26 (7.80, 16.72)	135.44 (95.21, 175.66)	15.44 (11.17, 19.70)	0.91 (0.78, 1.04)*	16.98 (14.99, 18.96)	183.02 (140.37, 225.67)
Male > = 50	145	133.97 (76.70, 191.23)	2.92 (1.00, 4.85)	23.46 (10.39, 36.54)	281.62 (209.45, 353.79)	12.90 (8.72, 17.07)	1.54 (1.24, 1.83)	26.70 (22.99, 30.41)	349.15 (276.38, 421.91)

Means (95% CIs) are given from a weighted analysis. *P*-values were given from weighted multiple linear regression.

***P < 0.001 compared with non-Hispanic white participants.

**0.001 < = *P* < 0.01 compared with non-Hispanic white participants.

*0.01 < = *P* < 0.05 compared with non-Hispanic white participants.

### 3.3 Associations between dietary flavonoid intake and the risk of prediabetes

**FIGURE 2:**
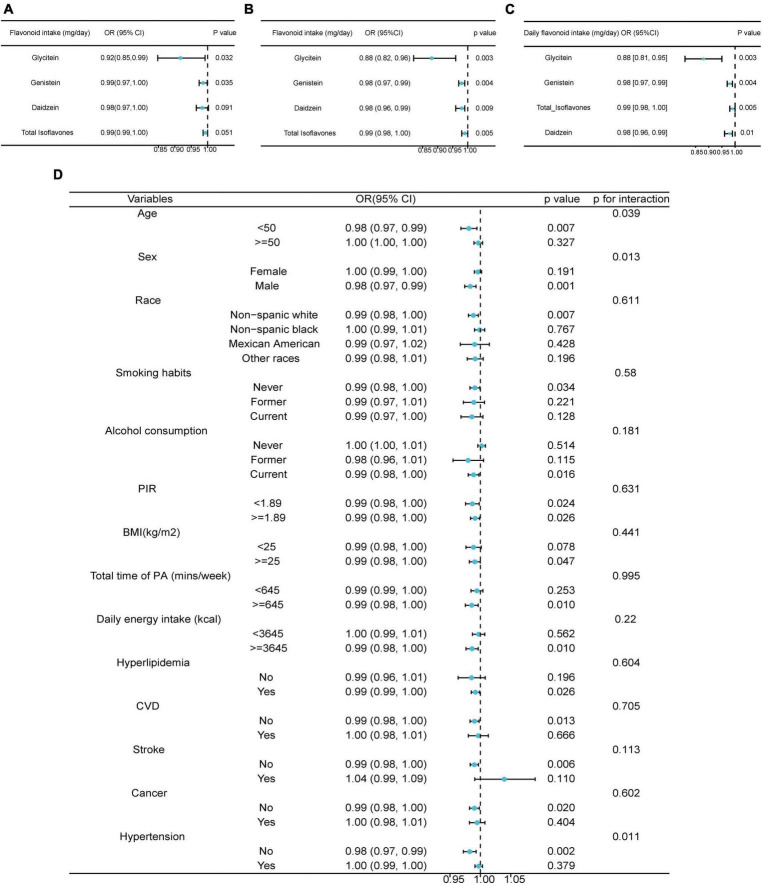
Associations between dietary flavonoid intake and the risk of prediabetes. **(A)** The univariate weighted Logistics regression analyses on the association between dietary flavonoid intake and the risk of prediabetes. **(B)** The multivariate weighted Logistics regression analyses adjusted for age, sex, race, BMI, daily energy intake, total time of PA, smoking habits, alcohol consumption, hyperlipidemia, and hypertension. **(C)** The multivariate weighted Logistics regression analyses adjusted for age, sex, race, BMI, daily energy intake, total time of PA, smoking habits, alcohol consumption, hyperlipidemia, hypertension, and survey year cycle. **(D)** Stratified analysis on the association between dietary flavonoid intake and the risk of prediabetes with adjustment for age, sex, race, BMI, daily energy intake, total time of PA, smoking habits, alcohol consumption, hyperlipidemia, and hypertension. BMI, body mass index; PA, physical activity; CVD, cardiovascular disease; PIR, poverty income ratio.

The association between the risk of prediabetes and the intake of all available individual flavonoids and subclasses was evaluated using weighted logistic regression analyses (Figures 2A–C; Supplementary Figures 1–3). The unadjusted logistic analysis demonstrated positive associations between the intake of certain flavonoids including catechin, theaflavin, thearubigins, isorhamnetin, kaempferol, myricetin, quercetin, theaflavin-3,3‘-digallate, theaflavin-3′-gallate, theaflavin-3-gallate, gallocatechin, total flavan 3-ols, total flavonols, total flavonoids, epigallocatechin, epicatechin 3-gallate, luteolin, subtotal catechins, epicatechin, and epigallocatechin 3-gallate with the risk of prediabetes (Supplementary Figure 1). The consumptions of glycitein, genistein, and pelargonidin were inversely associated with the risk of prediabetes in the unadjusted logistic regression (Figure 2A; Supplementary Figure 1). Notably, the intake of glycitein (OR: 0.88; 95% CI: 0.82–0.96; *p* = 0.003) and genistein (OR: 0.98; 95% CI: 0.97–0.99; *p* = 0.004) remained inversely associated with the risk of prediabetes after full adjustment for age, sex, race, BMI, daily energy intake, total time of PA smoking habits, alcohol consumption, hyperlipidemia, and hypertension (Figure 2B). Moreover, the intakes of total isoflavones (OR: 0.99; 95% CI: 0.98–1.00; *p* = 0.005) and daidzein (OR: 0.98, 95% CI: 0.96–0.99; *p* = 0.009) were inversely associated with the risk of prediabetes after full adjustment (Figure 2B). The positive association between the intake of myricetin (OR: 1.03; 95% CI: 1.00–1.06; *p* = 0.041) and the risk of prediabetes remained after full adjustment (Supplementary Figure 2). Upon adjusting for survey year cycles as a covariate, the inverse correlations between glycitein, genistein, isoflavones, daidzein, and prediabetes remained unaffected (Figure 2C; Supplementary Figure 3).

The heterogeneity among subgroups was then explored. The differences in the isoflavone effects among age, sex and hypertension history were significant (p for interaction = 0.039, p for interaction = 0.013, p for interaction = 0.011) (Figure 2D). Specifically, the intake of isoflavone was inversely associated with the risk of prediabetes in males and in the participants without hypertension (OR: 0.98; 95% CI: 0.97–0.99; *p* = 0.001; 0.98; 95% CI: 0.97–0.99; *p* = 0.032, respectively) (Figure 2D).

Furthermore, the inverse associations between the risk of prediabetes and intake of isoflavones were observed in the participants younger than 50, non-Hispanic white participants, non-smokers, current drinkers, population with BMI > = 25, participants with more time of PA (> = 645 min/week), people with daily energy intake > = 3,645 kcal, participants with hyperlipidemia, without CVD, without stroke, or cancer (Figure 2D). The PIR did not influence the negative correlation between isoflavone consumption and the risk of prediabetes (Figure 2D).

### 3.4 Association between urinary isoflavone metabolites and prediabetes risk

The estimation of flavonoid intake is subject to diverse factors, such as wide variations of flavonoid concentrations in foods and complex metabolic processes, thus urinary isoflavones are regarded as an objective indicative measure for assessing the intake, absorption, and metabolism of these compounds (27). Hence, we conducted a weighted analysis to evaluate the correlation between urinary levels of isoflavones and the occurrence of prediabetes. A total of 3,706 participants with or without prediabetes with complete records of urinary isoflavones from NHANES 2007–2010 were acquired in this analysis, which could represent 192,302,378 non-institutionalized US population.

The baseline characteristics of the study cohort in accordance with the quartiles of urinary daidzein were shown in Supplementary Table 3. At the fourth quartile of urinary daidzein concentrations compared to the first quartile, participants were younger (*p* < 0.001), and at the third quartile of urinary daidzein concentrations, participants had the lowest HEI score (*p* = 0.03) (Supplementary Table 3). At the second quartile of urinary daidzein concentration, participants had the highest risk of CVD (*p* = 0.01) (Supplementary Table 3). As urinary daidzein concentrations progressively increased, urinary concentrations of O-desmethylangolensin, equol, enterodiol, enterolactone, genistein, and creatinine increased (Supplementary Table 3).

Firstly, we investigated the association between metabolite concentration in urine as a continuous variable and prediabetes risk. As the concentrations of metabolites in urine are not normally distributed, a log10 transformation on the urinary metabolite concentrations were conducted. After fully adjusting for age, sex, race, BMI, daily energy intake, total time of PA, smoking habits, alcohol consumption, hyperlipidemia, hypertension, and creatinine, the concentrations of daidzein and genistein in urine were found to have a negative correlation with prediabetes risk (OR: 0.84, 95% CI: 0.73–0.96, *p* = 0.012; OR: 0.83, 95% CI: 0.75–0.93; *p* = 0.003, respectively, Table 3). However, O-Desmethylangolensin, equol, enterodiol, and enterolactone showed no significant association with prediabetes risk (Table 3; Supplementary Table 4). To further confirm this association, the urine metabolite concentrations were divided into quartiles. Notably, the urinary concentration of daidzein and genistein was inversely associated with the risk of prediabetes after fully adjustment (p for trend = 0.015, p for trend = 0.026, Supplementary Table 5), which is consistent with the inverse association between prediabetes risk and dietary intake of daidzein and genistein. In addition, the urinary concentration of O-desmethylangolensin, equol, enterodiol, and enterolactone was not associated with the risk of prediabetes (Supplementary Table 5).

**TABLE 3:** The correlation between urinary isoflavone metabolites and prediabetes risk.

Urinary concentration (log_10_, ng/ml)		OR (95% CI)	*p*-value
**Daidzein**			
	Model 1	0.83 (0.73, 0.94)	0.006
	Model 2	0.83 (0.73, 0.95)	0.009
	Model 3	0.84 (0.73, 0.96)	0.012
**Genistein**			
	Model 1	0.85 (0.77, 0.94)	0.003
	Model 2	0.85 (0.76, 0.94)	0.003
	Model 3	0.83 (0.75, 0.93)	0.003
**Equol**			
	Model 1	0.85 (0.75, 0.97)	0.016
	Model 2	0.91 (0.76, 1.08)	0.250
	Model 3	0.92 (0.77, 1.10)	0.358
**O-Desmethylangolensin**			
	Model 1	0.93 (0.83, 1.03)	0.163
	Model 2	0.96 (0.84, 1.08)	0.456
	Model 3	0.98 (0.87, 1.11)	0.732

The concentrations of urinary isoflavone metabolites were transformed by taking the logarithm (base 10). Model 1 was the crude model. Model 2 was adjusted for age, sex, race, BMI, and creatinine. Model 3 was adjusted for age, sex, race, BMI, daily energy intake (kcal/day), total of time of PA, smoking habits, alcohol consumption, hyperlipidemia history, hypertension history and urinary creatinine. Ref. reference.

Moreover, a stratified analysis was conducted. In individuals aged 50 years and above, non-Hispanic white participants, non-smokers, current drinkers, with a daily intake of ≥3,828 kcal, and with hyperlipidemia, the concentrations of daidzein and genistein in urine were found to exhibit an inverse association with the risk of prediabetes (Supplementary Figures 4, 5). Additionally, in women and participants with no history of hypertension, the concentration of daidzein in urine was found to be negatively correlated with the risk of prediabetes (Supplementary Figure 4). In participants with a BMI greater than or equal to 25.36 and a weekly exercise time of less than 620 min, the concentration of genistein in urine was found to exhibit a negative correlation with the risk of prediabetes (Supplementary Figure 5). The concentration of genistein in urine was found to show a negative correlation with the risk of prediabetes, regardless of participants’ hypertension status (Supplementary Figure 5).

## 4 Discussion

Our findings reveal an inverse association between dietary isoflavones and the risk of prediabetes, as well as an inverse association between urinary levels of isoflavones and the risk of prediabetes. In our previous study, we assessed the correlation between flavonoid intake and the risk of DM in the NHANES cohort but did not find any significant association with isoflavone intake (OR: 1.01; 95% CI: 0.86–1.19; *p* = 0.872) (16). In the current study, we refined our study population by excluding individuals diagnosed with DM and focused specifically on individuals with prediabetes, who were previously considered to be part of the healthy population (16). By studying this early stage of abnormal glucose metabolism, our study fills a research gap in understanding the correlation between isoflavones and the risk of prediabetes. The mechanisms underlying the relationship between isoflavones and the reduced risk of prediabetes are not yet comprehensively understood, although various potential mechanisms have been proposed. Consumption of soy and soy products has been associated with the preservation of β-cells in pancreatic islets (28). Genistein and/or daidzein exhibit inhibitory effects on α-glucosidase, along with increased secretion of insulin *in vitro* (29, 30), and *in vivo* (31–34), activation of hepatic glucokinase, suppression of gluconeogenic enzyme synthesis (31, 35), and inhibition of glucose uptake in adipocyte cells (36). Additionally, isoflavones can be transformed into equol or a ring-fission product, O-desmethylangolensin by microbes in the intestine. Compared to daidzein, equol demonstrates a higher affinity to oestrogen receptors, stronger antioxidant efficacy, and exhibits anti-androgenic properties (37). The main sources of isoflavone are soy and soy products in humans. In the US, the consumption of soybeans and soy products is approximately one-tenth of that observed in Asian countries (38). Despite the relatively low intake, our study still identified significantly inverse associations between prediabetes risk and dietary daidzein, glycitein, genistein, and total isoflavones in the US. The available literature on the relationship between intake of soy or isoflavone and prediabetes is limited. The association between the intake of soy or isoflavones and T2D incidence is controversial. The risk of T2D is negatively related to soy intake in middle-aged Chinese women (39). The inverse association between isoflavones intake and T2D risk was observed in the US population (40). However, in other reports, consumption of soy products was linked to a mildly increased risk of T2D (41). Dietary isoflavone consumption was marginally significantly negatively associated with the risk of T2D (42). Dietary isoflavones were not associated with the risk of T2D in Europe population (43) and in the NHANES cohort (16). In Japanese, the intakes of soy products and isoflavones were not significantly associated with T2D, except the overweight women (44). However, in our study, there was no difference observed in the association between isoflavones and prediabetes across different racial/ethnic groups. Those conflicting results may be due to variations in the food items assessed in the questionnaires, divergent dietary patterns among the cohorts, and disparities in the databases employed for evaluating flavonoid consumption. Apart from potential estimation inaccuracies, there is considerable interindividual variability in flavonoid metabolism (45).

To further confirm the inverse correlation between flavonoids and prediabetes risk, we used the urinary concentration of isoflavones to further explore the role of isoflavones in the onset of prediabetes. Our further analysis found a negative correlation between urinary daidzein and genistein concentrations and prediabetes risk. Urinary isoflavones and their metabolites are considered to be more objective and measurable indicators that allow better screening of the correlation between isoflavones and glycemic control. In pregnant women, urinary isoflavones were found to be negatively correlated with fasting glucose, insulin, and the Homeostatic Model Assessment of Insulin Resistance (HOMA-IR) (46). However, more researches revealed inconsistent results. Soy consumption was not associated with the risk of glycosuria (47). Urinary isoflavones and metabolites were found to have no association with the risk of T2D (48). No association between urinary concentrations of total isoflavones and their metabolites and risk of T2D in Chinese adults (48). We demonstrated an inverse association between isoflavones and the risk of prediabetes at both the dietary intake and urine concentration. Our findings may suggest that the optimal protective effect of isoflavones may be most pronounced in individuals with mild pancreatic impairment, such as prediabetes. Considering the implications of our study findings for clinical practice or research, we suggest that investigations into the isoflavones should be prioritized in individuals with prediabetes or the population without diagnosed diabetes but with risk factors for developing diabetes. Our results may provide preliminary explanation regarding the conflicting results from the association between T2D risk and dietary isoflavones.

While equol is considered the active form of soy isoflavones and possesses a more potent estrogenic-like effect compared to daidzein (49), we did not find that urinary equol was associated the risk of prediabetes. The result may suggest that the mechanism of isoflavone in regulating blood glucose homeostasis is independent of oestrogen-like activity of isoflavones. Non-oestrogen-like effects of isoflavones influencing glucose homeostasis may be through numerous other mechanisms, such as the antioxidant activity and anti-inflammatory properties. modulation of Ca^2 +^ signaling and cAMP/protein kinase A (AMPK) function (32).

Additionally, the myricetin intake did not associate with a decreased likelihood of T2D (50, 51). However, we found that the intake of myricetin was significantly positively associated with prediabetes. More research is needed. Enterolactone and enterodiol are derivatives of lignan from sesame seed or flaxseed. We also did not observe a negative correlation between the enterolactone and enterodiol concentration and the risk of prediabetes. Our results are consistent with the findings of the previous study conducted in the Singapore Chinese (48).

Our study is limited by the lack of consideration for potential changes in dietary patterns throughout the follow-up period. The data on dietary intake and metabolites in urine were only gathered from the initial questionnaire and examination. Moreover, our study findings do not allow for attributing the potential beneficial effects to the entire subclass of isoflavones. Only a limited number of molecules, namely, daidzein and genistein, demonstrated significant associations with a reduced risk of prediabetes, and the observed protective effect was modest. Additionally, it is crucial to acknowledge that observational studies are incapable of establishing a cause-and-effect relationship.

## 5 Conclusion

To conclude, our study outcomes indicate an inverse association between dietary isoflavone consumption and the risk of prediabetes. Moreover, the urinary daidzein and genistein levels were inversely associated with the risk of prediabetes. Our study highlights the significance of employing diverse indicators or measurements for evaluating the health impacts of isoflavones. Our research findings suggested that the application of isoflavones should be initiated earlier in the prevention and management of diabetes.

## Data availability statement

Publicly available datasets were analyzed in this study. This data can be found here: https://www.cdc.gov/nchs/nhanes (accessed on 4 July 2023).

## Ethics statement

The studies involving humans were approved by the Institutional Review Board (IRB) or Ethics Committee of the National Center for Health Statistics, CDC (Protocol #2005-06, #2011-17, #2018-01). The studies were conducted in accordance with the local legislation and institutional requirements. The participants provided their written informed consent to participate in this study.

## Author contributions

YJZ: Conceptualization, Data curation, Formal analysis, Funding acquisition, Investigation, Methodology, Project administration, Resources, Software, Supervision, Validation, Visualization, Writing—original draft, Writing—review and editing. SQ: Validation, Visualization, Writing—original draft. YZ: Visualization, Writing—original draft. PX: Conceptualization, Formal analysis, Methodology, Project administration, Supervision, Writing—original draft, Writing—review and editing. KG: Funding acquisition, Writing—original draft.
