# Supplementary material for: Inverse association between isoflavones and prediabetes risk: evidence from NHANES 2007–2010 and 2017–2018
Source: Front Nutr. 2023 Dec 5;10:1288416. doi: 10.3389/fnut.2023.1288416 (PMC10728643; doi:10.3389/fnut.2023.1288416)
Supplement: Supplementary file 1 [file Data_Sheet_1.docx]

Supplementary Material

# Supplementary Tables

**Supplementary Table 1.** The main subclasses of flavonoids and relative members.

| Subclass | Member |
| --- | --- |
| total anthocyanidins | cyanidin |
|  | delphinidin |
|  | malvidin |
|  | pelargonidin |
|  | peonidin |
|  | petunidin |
| subtotal catechins | (-)-epicatechin |
|  | (-)-epicatechin 3-gallate |
|  | (-)-epigallocatechin |
|  | (-)-epigallocatechin 3-gallate |
|  | (+)-catechin |
|  | (+)-gallocatechin |
| total flavan-3-ol | (-)-epicatechin |
|  | (-)-epicatechin 3-gallate |
|  | (-)-epigallocatechin |
|  | (-)-epigallocatechin 3-gallate |
|  | (+)-catechin |
|  | (+)-gallocatechin |
|  | theaflavin |
|  | theaflavin-3,3’-digallate |
|  | theaflavin-3’-gallate |
|  | theaflavin-3-gallate |
|  | thearubigins |
| total flavanones | eriodictyol |
|  | hesperetin |
|  | naringenin |
| total flavones | apigenin |
|  | luteolin |
| total flavonols | isohamnetin |
|  | kaempferol |
|  | myricetin |
|  | quercetin |
| total isoflavones | diadzein |
|  | genistein |
|  | glycitein |

**Supplementary Table 2.** Characteristics of participants by survey year cycles, NHANES 2007-2008, 2009-2010, and 2017–2018.

| **Variables** | **2007-2008** | **2009-2010** | **2017-2018** | **p value** | **p value for 2007 vs 2009** | **p value for 2007 vs 2017** | **p value for 2009 vs 2017** |  |  |  |  |
| --- | --- | --- | --- | --- | --- | --- | --- | --- | --- | --- | --- |
| **Baseline sociodemographic, lifestyle, and health-related variables** | | | | |  |  |  |  |  |  |  |
| Age (years) | 34.74(0.62) | 34.96(0.53) | 36.21(0.42) | 0.08 | 0.79 | 0.06 | 0.07 |  |  |  |  |
| Sex |  |  |  | 0.36 | 0.11 | 0.27 | 0.92 |  |  |  |  |
| Female | 3308(53.26%) | 3637(51.68%) | 2822(51.53%) |  |  |  |  |  |  |  |  |
| Male | 3204(46.74%) | 3461(48.32%) | 2589(48.47%) |  |  |  |  |  |  |  |  |
| Race |  |  |  | 0.12 | 0.71 | 0.04 | 0.23 |  |  |  |  |
| Non-Hispanic black participants | 1398(11.78%) | 1241(11.70%) | 1314(11.71%) |  |  |  |  |  |  |  |  |
| Mexican American | 1320(9.91%) | 1538(9.91%) | 752(11.28%) |  |  |  |  |  |  |  |  |
| Other Races | 254(4.96%) | 423(6.84%) | 987(10.84%) |  |  |  |  |  |  |  |  |
| Non-Hispanic white participants | 3540(73.34%) | 3896(71.55%) | 2358(66.18%) |  |  |  |  |  |  |  |  |
| Academic level |  |  |  | 0.005 | 0.14 | 0.003 | 0.04 |  |  |  |  |
| <9 years | 542(5.30%) | 579(4.72%) | 268(2.73%) |  |  |  |  |  |  |  |  |
| 9-12 years | 2677(37.62%) | 2676(34.07%) | 1850(32.87%) |  |  |  |  |  |  |  |  |
| >12 years | 3293(57.09%) | 3843(61.21%) | 3293(64.40%) |  |  |  |  |  |  |  |  |
| Partner status |  |  |  | 0.57 | 0.52 | 0.37 | 0.7 |  |  |  |  |
| Without partner | 3484(48.37%) | 3808(47.18%) | 2826(46.64%) |  |  |  |  |  |  |  |  |
| With partner | 3028(51.63%) | 3290(52.82%) | 2585(53.36%) |  |  |  |  |  |  |  |  |
| Smoking habits |  |  |  | 0.003 | 0.08 | < 0.001 | 0.15 |  |  |  |  |
| Never | 4143(61.24%) | 4701(64.27%) | 3734(66.98%) |  |  |  |  |  |  |  |  |
| Former | 1125(18.18%) | 1162(18.51%) | 860(18.31%) |  |  |  |  |  |  |  |  |
| Current | 1244(20.58%) | 1235(17.22%) | 817(14.71%) |  |  |  |  |  |  |  |  |
| Alcohol consumption |  |  |  | < 0.0001 | 0.14 | < 0.0001 | < 0.0001 |  |  |  |  |
| Never | 873(11.19%) | 820(10.02%) | 611(9.00%) |  |  |  |  |  |  |  |  |
| Former | 925(13.41%) | 843(11.15%) | 67(0.85%) |  |  |  |  |  |  |  |  |
| Current | 4714(75.40%) | 5435(78.83%) | 4733(90.15%) |  |  |  |  |  |  |  |  |
| PIR | 2.90(0.10) | 2.85(0.06) | 2.88(0.07) | 0.87 | 0.63 | 0.86 | 0.7 |  |  |  |  |
| BMI (kg/m^2^) | 25.80(0.14) | 25.91(0.14) | 26.83(0.20) | < 0.001 | 0.56 | < 0.001 | < 0.001 |  |  |  |  |
| HEI score of 2015 | 51.69(0.65) | 53.03(0.38) | 51.74(0.63) | 0.09 | 0.08 | 0.95 | 0.09 |  |  |  |  |
| DII | 1.84(0.09) | 1.51(0.04) | 1.62(0.07) | 0.01 | 0.003 | 0.07 | 0.2 |  |  |  |  |
| Total time of PA (mins/week) | 1271.76(43.38) | 1077.54(37.62) | 1470.17(51.64) | < 0.0001 | 0.002 | 0.01 | < 0.0001 |  |  |  |  |
| Total MET of PA (/week) | 5209.11(209.10) | 4302.25(197.50) | 6181.90(323.53) | < 0.0001 | 0.004 | 0.02 | < 0.0001 |  |  |  |  |
| Daily energy intake (kcal) | 4049.21(40.81) | 4134.42(40.98) | 4048.63(34.02) | 0.22 | 0.15 | 0.99 | 0.12 |  |  |  |  |
| **Disease history at interview** | | | | | | | |  |  |  |  |
| Hyperlipidaemia |  |  |  | 0.01 | 0.79 | 0.01 | 0.02 |  |  |  |  |
| No | 2427(40.03%) | 2678(40.43%) | 2308(45.83%) |  |  |  |  |  |  |  |  |
| Yes | 3212(59.97%) | 3491(59.57%) | 2498(54.17%) |  |  |  |  |  |  |  |  |
| CVD |  |  |  | 0.93 | 0.78 | 0.7 | 0.95 |  |  |  |  |
| No | 3438(93.80%) | 3799(94.05%) | 2973(94.11%) |  |  |  |  |  |  |  |  |
| Yes | 337(6.20%) | 336(5.95%) | 279(5.89%) |  |  |  |  |  |  |  |  |
| Stroke |  |  |  | 0.25 | 0.25 | 0.16 | 0.76 |  |  |  |  |
| No | 3640(97.28%) | 4011(97.80%) | 3134(97.89%) |  |  |  |  |  |  |  |  |
| Yes | 124(2.72%%) | 124(2.20%) | 117(2.11%) |  |  |  |  |  |  |  |  |
| Cancer |  |  |  | 0.63 | 0.57 | 0.3 | 0.76 |  |  |  |  |
| No | 3428(91.90%) | 3746(91.34%) | 2943(91.05%) |  |  |  |  |  |  |  |  |
| Yes | 342(8.10%) | 386(8.66%) | 309(8.95%) |  |  |  |  |  |  |  |  |
| Hypertension |  |  |  | 0.11 | 0.53 | 0.11 | 0.07 |  |  |  |  |
| No | 5099(77.63%) | 5664(78.74%) | 4060(75.06%) |  |  |  |  |  |  |  |  |
| Yes | 1413(22.37%) | 1434(21.26%) | 1351(24.94%) |  |  |  |  |  |  |  |  |
| **Intake of flavonoids (mg/day)** | | | | |  |  |  |  | Intake of flavonoids (mg/day) |  |  |
| Daidzein | 0.54(0.07) | 0.69(0.05) | 0.88(0.10) | 0.03 | 0.1 | 0.01 | 0.11 |  |  |  |  |
| Genistein | 0.72(0.09) | 0.97(0.06) | 1.27(0.15) | 0.01 | 0.03 | 0.004 | 0.07 |  |  |  |  |
| Glycitein | 0.09(0.01) | 0.13(0.01) | 0.20(0.02) | 0.001 | 0.01 | < 0.001 | 0.02 |  |  |  |  |
| Cyanidin | 2.02(0.20) | 2.68(0.17) | 2.59(0.36) | 0.05 | 0.02 | 0.17 | 0.82 |  |  |  |  |
| Petunidin | 0.70(0.10) | 1.18(0.16) | 1.26(0.16) | 0.004 | 0.02 | 0.01 | 0.72 |  |  |  |  |
| Delphinidin | 0.93(0.13) | 1.78(0.26) | 1.77(0.22) | < 0.001 | 0.01 | 0.002 | 0.97 |  |  |  |  |
| Malvidin | 3.85(0.41) | 4.71(0.44) | 4.87(0.48) | 0.21 | 0.17 | 0.12 | 0.81 |  |  |  |  |
| Pelargonidin | 1.48(0.25) | 1.91(0.34) | 1.81(0.17) | 0.47 | 0.32 | 0.27 | 0.81 |  |  |  |  |
| Peonidin | 1.12(0.10) | 1.76(0.25) | 2.45(0.32) | < 0.001 | 0.02 | < 0.001 | 0.1 |  |  |  |  |
| Catechin | 6.94(0.33) | 7.38(0.20) | 6.75(0.25) | 0.14 | 0.27 | 0.64 | 0.06 |  |  |  |  |
| Epigallocatechin | 13.88(0.98) | 13.06(1.15) | 12.34(0.71) | 0.45 | 0.59 | 0.21 | 0.6 |  |  |  |  |
| Epicatechin | 9.73(0.42) | 9.68(0.33) | 9.40(0.28) | 0.73 | 0.93 | 0.52 | 0.51 |  |  |  |  |
| Epicatechin 3-gallate | 9.15(0.66) | 8.55(0.78) | 7.64(0.47) | 0.17 | 0.57 | 0.07 | 0.33 |  |  |  |  |
| Epigallocatechin 3-gallate | 24.14(1.70) | 22.51(1.99) | 20.16(1.46) | 0.21 | 0.54 | 0.09 | 0.35 |  |  |  |  |
| Theaflavin | 1.39(0.10) | 1.30(0.13) | 1.14(0.13) | 0.36 | 0.61 | 0.16 | 0.4 |  |  |  |  |
| Thearubigins | 80.29(5.88) | 74.63(7.12) | 65.02(6.69) | 0.24 | 0.54 | 0.1 | 0.33 |  |  |  |  |
| Eriodictyol | 0.19(0.01) | 0.19(0.01) | 0.13(0.02) | 0.004 | 0.86 | 0.004 | 0.002 |  |  |  |  |
| Hesperetin | 9.85(0.56) | 10.25(0.39) | 7.38(0.41) | < 0.0001 | 0.56 | 0.001 | < 0.0001 |  |  |  |  |
| Naringenin | 3.21(0.23) | 3.30(0.20) | 3.07(0.26) | 0.78 | 0.79 | 0.68 | 0.49 |  |  |  |  |
| Apigenin | 0.19(0.02) | 0.26(0.06) | 0.14(0.01) | 0.03 | 0.27 | 0.06 | 0.05 |  |  |  |  |
| Luteolin | 0.56(0.04) | 0.64(0.03) | 0.64(0.03) | 0.17 | 0.08 | 0.12 | 0.98 |  |  |  |  |
| Isorhamnetin | 0.66(0.03) | 0.76(0.02) | 0.75(0.03) | 0.03 | 0.01 | 0.03 | 0.72 |  |  |  |  |
| Kaempferol | 3.73(0.18) | 3.86(0.14) | 3.97(0.13) | 0.52 | 0.55 | 0.26 | 0.57 |  |  |  |  |
| Myricetin | 1.23(0.07) | 1.21(0.06) | 1.33(0.05) | 0.26 | 0.82 | 0.24 | 0.14 |  |  |  |  |
| Quercetin | 10.12(0.43) | 10.40(0.26) | 9.31(0.24) | 0.01 | 0.57 | 0.11 | 0.004 |  |  |  |  |
| Theaflavin-3,3‘-digallate | 1.53(0.12) | 1.44(0.14) | 1.26(0.14) | 0.33 | 0.61 | 0.14 | 0.37 |  |  |  |  |
| Theaflavin-3’-gallate | 1.29(0.10) | 1.22(0.12) | 1.07(0.12) | 0.37 | 0.63 | 0.17 | 0.4 |  |  |  |  |
| Theaflavin-3-gallate | 1.11(0.08) | 1.04(0.10) | 0.89(0.10) | 0.27 | 0.6 | 0.11 | 0.32 |  |  |  |  |
| Gallocatechin | 1.47(0.11) | 1.40(0.12) | 1.20(0.09) | 0.13 | 0.66 | 0.06 | 0.19 |  |  |  |  |
| Subtotal Catechins | 65.31(4.03) | 62.58(4.49) | 57.49(2.97) | 0.28 | 0.65 | 0.13 | 0.35 |  |  |  |  |
| Total Isoflavones | 1.36(0.18) | 1.80(0.11) | 2.35(0.27) | 0.01 | 0.05 | 0.01 | 0.07 |  |  |  |  |
| Total Anthocyanidins | 10.09(0.91) | 14.01(0.80) | 14.75(1.37) | 0.003 | 0.003 | 0.01 | 0.64 |  |  |  |  |
| Total Flavan 3-ols | 150.92(10.30) | 142.21(12.09) | 126.86(8.53) | 0.19 | 0.59 | 0.08 | 0.31 |  |  |  |  |
| Total Flavanones | 13.25(0.76) | 13.73(0.58) | 10.57(0.62) | 0.002 | 0.62 | 0.01 | < 0.001 |  |  |  |  |
| Total Flavones | 0.74(0.05) | 0.89(0.07) | 0.77(0.04) | 0.19 | 0.08 | 0.64 | 0.13 |  |  |  |  |
| Total Flavonols | 15.73(0.68) | 16.24(0.45) | 15.37(0.40) | 0.36 | 0.54 | 0.65 | 0.16 |  |  |  |  |
| Total Sum of all 29 flavonoids | 192.10(11.31) | 188.88(12.49) | 170.68(9.34) | 0.29 | 0.85 | 0.15 | 0.25 |  |  |  |  |

The continuous variables were demonstrated as weighted mean (weighted standard error) and compared by Wilcoxon test, including age, PIR, score, HEI score, DII, BMI, total time of PA, and total MET of PA, daily energy intake, and the daily intake of flavonoids. The categorical variables were demonstrated as sample number (weighted percentage) and compared by chi-square test, including sex, ethnicity, education, partner status, smoking status, alcohol consumption, and disease history. PIR: poverty income ratio; HEI: healthy eating index; DII: dietary inflammatory index; BMI: Body mass index; PA: Physical Activity; MET: metabolic equivalent.

**Supplementary Table 3.** The baseline characteristics of the study population according to the quartiles of urinary daidzein.

|  | Concentration of urinary Daizen (ng/ml) | | | | |
| --- | --- | --- | --- | --- | --- |
| variable | Q1 | Q2 | Q3 | Q4 | p value |
| Baseline sociodemographic, lifestyle, and health-related variables | | | | | |
| Age | 40.97(0.80) | 37.58(1.21) | 36.48(0.81) | 36.74(0.83) | < 0.0001 |
| Sex |  |  |  |  | 0.44 |
| Female | 512(55.12%) | 478(51.53%) | 467(50.14%) | 466(52.44%) |  |
| Male | 415(44.88%) | 449(48.47%) | 462(49.86%) | 457(47.56%) |  |
| Race |  |  |  |  | 0.14 |
| Non-Hispanic black participants | 148(9.56%) | 169(10.35%) | 212(12.94%) | 202(11.38%) |  |
| Mexican American | 220(11.02%) | 201(9.75%) | 163(7.73%) | 205(9.83%) |  |
| Other Races | 42(6.50%) | 35(4.98%) | 49(6.40%) | 56(7.68%) |  |
| Non-Hispanic white participants | 517(72.92%) | 522(74.93%) | 505(72.94%) | 460(71.11%) |  |
| Academic level |  |  |  |  | 0.05 |
| <9 years | 110(6.33%) | 68(4.78%) | 74(4.96%) | 68(4.35%) |  |
| 9-12 years | 379(39.04%) | 380(35.76%) | 384(38.02%) | 366(33.27%) |  |
| >12 years | 438(54.63%) | 479(59.46%) | 471(57.02%) | 489(62.38%) |  |
| Partner status |  |  |  |  | 0.36 |
| without partner | 422(40.49%) | 484(43.88%) | 455(44.34%) | 494(45.75%) |  |
| with partner | 505(59.51%) | 443(56.12%) | 474(55.66%) | 429(54.25%) |  |
| Smoking habits |  |  |  |  | 0.31 |
| Never | 545(55.47%) | 566(59.86%) | 571(62.44%) | 583(61.72%) |  |
| Former | 190(22.74%) | 178(20.63%) | 172(19.80%) | 166(19.86%) |  |
| Current | 192(21.79%) | 183(19.51%) | 186(17.76%) | 174(18.42%) |  |
| Alcohol consumption |  |  |  |  | 0.59 |
| Never | 99(9.06%) | 113(9.10%) | 111(9.51%) | 119(11.51%) |  |
| Former | 112(11.39%) | 123(11.78%) | 112(10.87%) | 128(12.67%) |  |
| Current | 716(79.55%) | 691(79.12%) | 706(79.63%) | 676(75.82%) |  |
| PIR | 2.94(0.07) | 3.01(0.10) | 2.91(0.09) | 3.04(0.09) | 0.32 |
| BMI (kg/m2) | 26.52(0.20) | 26.63(0.36) | 26.81(0.21) | 25.92(0.30) | 0.11 |
| HEI score of 2015 | 53.31(0.70) | 51.33(0.61) | 51.07(0.59) | 52.97(0.65) | 0.03 |
| DII | 1.52(0.09) | 1.68(0.10) | 1.77(0.09) | 1.53(0.09) | 0.08 |
| Total time of PA (mins/week) | 1160.68(61.83) | 1197.13(67.31) | 1268.47(82.26) | 1259.15(55.98) | 0.38 |
| Daily energy intake (kcal) | 4151.30(64.21) | 4303.59(87.86) | 4092.15(54.39) | 4329.23(77.25) | 0.08 |
| Disease history at interview | | | | | |
| Hyperlipidaemia |  |  |  |  | 0.32 |
| No | 345(35.94%) | 387(40.09%) | 419(38.55%) | 438(41.08%) |  |
| Yes | 582(64.06%) | 540(59.91%) | 510(61.45%) | 485(58.92%) |  |
| CVD |  |  |  |  | 0.01 |
| No | 689(96.31%) | 560(91.55%) | 527(94.20%) | 507(94.43%) |  |
| Yes | 33(3.69%) | 56(8.45%) | 52(5.80%) | 44(5.57%) |  |
| Respiratory diseases |  |  |  |  | 0.25 |
| Asthma and COPD | 13(2.18%) | 13(1.63%) | 16(1.82%) | 11(0.93%) |  |
| Asthma | 77(11.21%) | 61(8.64%) | 71(13.78%) | 76(12.92%) |  |
| COPD | 34(5.31%) | 23(3.08%) | 27(4.87%) | 19(3.93%) |  |
| No | 608(81.30%) | 544(86.66%) | 484(79.53%) | 463(82.22%) |  |
| Stroke |  |  |  |  | 0.4 |
| No | 709(98.19%) | 595(96.88%) | 562(97.99%) | 539(98.52%) |  |
| Yes | 13(1.81%) | 20(3.12%) | 15(2.01%) | 12(1.48%) |  |
| Cancer |  |  |  |  | 0.32 |
| No | 673(92.96%) | 551(89.34%) | 514(89.99%) | 501(91.21%) |  |
| Yes | 49(7.04%) | 65(10.66%) | 64(10.01%) | 48(8.79%) |  |
| Hypertension |  |  |  |  | 0.54 |
| No | 675(73.86%) | 696(75.77%) | 728(77.21%) | 724(77.73%) |  |
| Yes | 252(26.14%) | 231(24.23%) | 201(22.79%) | 199(22.27%) |  |
| Concentration in urine (ng/ml) | | | | | |
| Diadzein | 9.59(0.27) | 34.02(0.50) | 118.39(2.29) | 1807.07(152.84) | < 0.0001 |
| O-desmethylangolensin | 1.85(0.21) | 7.51(0.90) | 26.50(2.55) | 471.08(62.28) | < 0.0001 |
| Equol | 11.37(2.04) | 16.90(1.58) | 61.59(17.22) | 223.35(55.80) | < 0.001 |
| Enterodiol | 86.43(8.85) | 111.62(14.34) | 176.96(35.66) | 175.55(19.19) | < 0.001 |
| Enterolactone | 461.31(36.77) | 625.64(50.22) | 896.85(129.95) | 1043.68(108.17) | < 0.0001 |
| Genistein | 10.79(1.79) | 24.79(2.98) | 69.36(4.49) | 762.64(71.70) | < 0.0001 |
| Creatinine | 85.77(3.18) | 124.76(3.46) | 132.82(3.74) | 141.93(3.51) | < 0.0001 |

**Supplementary Table 4.** The Associations between urinary metabolites and prediabetes risk by weighted Logistic regression.

| Urinary metabolites (log_10_, ng/ml) | OR (95% CI) | p value |
| --- | --- | --- |
| Enterodiol | 1.00 (1.00, 1.00) | 0.377 |
| Enterolactone | 1.00 (1.00, 1.00) | 0.213 |

The concentrations of urinary isoflavone metabolites were transformed by taking the logarithm (base 10). Model was adjusted for age, sex, race, BMI, total of time of PA, smoking habits, alcohol consumption, hyperlipidaemia history, hypertension history and urinary creatinine. BMI body mass index, PA physical activity.

**Supplementary Table 5.** The correlation between the concentrations of metabolites in urine and the prediabetes risk.

| Variables |  | Q1 | Q2 | | Q3 | | Q4 | | p for trend (Median value) |
| --- | --- | --- | --- | --- | --- | --- | --- | --- | --- |
|  |  |  | OR (95%CI) | p value | OR (95%CI) | p value | OR (95%CI) | p value |  |
| Diadzein (ng/ml) |  | [0.3,18.325] | (18.325,58.2] | | (58.2,219] | | (219,33000] |  |  |
| N (percentage) |  | 927(25.01%) | 927(25.01%) | | 929(25.07%) | | 923(24.91%) |  |  |
|  | Model 1 | Ref. | 0.83 (0.66, 1.04) | 0.096 | 0.85 (0.67, 1.07) | 0.15 | 0.66 (0.51, 0.85) | 0.002 | 0.005 |
|  | Model 2 | Ref. | 0.84 (0.62, 1.12) | 0.213 | 0.89 (0.67, 1.18) | 0.384 | 0.69 (0.52, 0.92) | 0.013 | 0.015 |
|  | Model 3 | Ref. | 0.84 (0.62, 1.14) | 0.249 | 0.89 (0.67, 1.20) | 0.437 | 0.69 (0.51, 0.92) | 0.016 | 0.015 |
| Genistein (ng/ml) |  | [0.1,9.2] | (9.2,26.65] | | (26.65,98.725] | | (98.725,16000] |  |  |
| N (percentage) |  | 932 (25.15%) | 921 (24.85%) | | 926 (24.99%) | | 927 (25.01%) |  |  |
|  | Model 1 | Ref. | 0.84 [0.67, 1.06] | 0.128 | 0.99 [0.83, 1.18] | 0.905 | 0.74 [0.58, 0.93] | 0.011 | 0.028 |
|  | Model 2 | Ref. | 0.90 [0.67, 1.20] | 0.45 | 1.04 [0.83, 1.32] | 0.703 | 0.74 [0.57, 0.97] | 0.032 | 0.026 |
|  | Model 3 | Ref. | 0.89 (0.67, 1.18) | 0.391 | 1.02 (0.79, 1.30) | 0.88 | 0.72 (0.54, 0.97) | 0.03 | 0.026 |
| Equol (ng/ml) |  | [0.04,3.12] | (3.12,7.115] | | (7.115,15.6] | | (15.6,17400] |  |  |
| N (percentage) |  | 928 (25.04%) | 925 (24.96%) | | 931 (25.12%) | | 922 (24.88%) |  |  |
|  | Model 1 | Ref. | 0.92 (0.70, 1.20) | 0.522 | 0.83 (0.67, 1.04) | 0.106 | 0.69 (0.55, 0.86) | 0.002 | <0.001 |
|  | Model 2 | Ref. | 0.96 (0.74, 1.25) | 0.771 | 0.81 (0.61, 1.08) | 0.137 | 0.71 (0.51, 0.99) | 0.047 | 0.03 |
|  | Model 3 | Ref. | 0.96 (0.72, 1.27) | 0.749 | 0.84 (0.62, 1.12) | 0.214 | 0.75 (0.52, 1.07) | 0.107 | 0.074 |
| O-Desmethylangolensin (ng/ml) | | [0.1,0.7] | (0.7,3.7] | | (3.7,21.975] | | (21.975,21600] |  |  |
| N (percentage) |  | 936 (25.26%) | 924 (24.93%) | | 919 (24.8%) | | 927 (25.01%) |  |  |
|  | Model 1 | Ref. | 0.90 (0.70, 1.15) | 0.373 | 0.83 (0.64, 1.07) | 0.137 | 0.78 (0.57, 1.06) | 0.104 | 0.182 |
|  | Model 2 | Ref. | 0.85 (0.65, 1.11) | 0.22 | 0.84 (0.64, 1.11) | 0.208 | 0.87 (0.61, 1.22) | 0.398 | 0.747 |
|  | Model 3 | Ref. | 0.86 (0.66, 1.13) | 0.263 | 0.90 (0.67, 1.20) | 0.436 | 0.92 (0.64, 1.32) | 0.635 | 0.987 |
| Enterodiol (ng/ml) |  | [0.03,14.3] | (14.3,39.95] | | (39.95,98.35] | | (98.35,13500] |  |  |
| N (percentage) |  | 929 (25.07%) | 924 (24.93%) | | 926 (24.99%) | | 927 (25.01%) |  |  |
|  | Model 1 | Ref. | 1.07 (0.81, 1.41) | 0.617 | 1.02 (0.77, 1.36) | 0.869 | 1.06 (0.75, 1.50) | 0.728 | 0.82 |
|  | Model 2 | Ref. | 1.00 (0.70, 1.44) | 0.981 | 0.98 (0.72, 1.34) | 0.91 | 1.04 (0.66, 1.62) | 0.874 | 0.833 |
|  | Model 3 | Ref. | 1.01 (0.70, 1.46) | 0.967 | 1.00 (0.72, 1.38) | 0.986 | 1.10 (0.69, 1.76) | 0.671 | 0.579 |
| Enterolactone (ng/ml) |  | [0.1,86.15] | (86.15,299] | | (299,776.75] | | (776.75,38000] |  |  |
| N (percentage) |  | 928 (25.04%) | (927) 25.01% | | 924 (24.93%) | | 927 (25.01%) |  |  |
|  | Model 1 | Ref. | 0.98 (0.75, 1.30) | 0.907 | 1.04 (0.80, 1.36) | 0.743 | 0.89 (0.66, 1.19) | 0.416 | 0.317 |
|  | Model 2 | Ref. | 1.03 (0.78, 1.38) | 0.806 | 0.95 (0.73, 1.24) | 0.697 | 0.76 (0.56, 1.04) | 0.088 | 0.027 |
|  | Model 3 | Ref. | 1.07 (0.79, 1.45) | 0.648 | 1.02 (0.78, 1.33) | 0.872 | 0.84 (0.61, 1.17) | 0.278 | 0.107 |

The concentrations of urinary metabolites were categorized into quartiles (Q1: 0-25%, Q2: 25%-50%, Q3: 50%-75%, Q4: 75%-100%) for Logistic regression analysis. Q1 was used as the reference category, and comparisons were made between Q2, Q3, and Q4 with Q1. The p-values for trend were determined to assess the statistical significance of a trend across ordered quartiles, where the median values of each quartile are utilized. Model 1 was the crude model. Model 2 was adjusted for age, sex, race, BMI, and creatinine. Model 3 was adjusted for age, sex, race, BMI, daily energy intake (kcal/day), total of time of PA, smoking habits, alcohol consumption, hyperlipidaemia history, hypertension history and urinary creatinine. Ref. reference; N the number of parcipants.

# Supplementary Figures


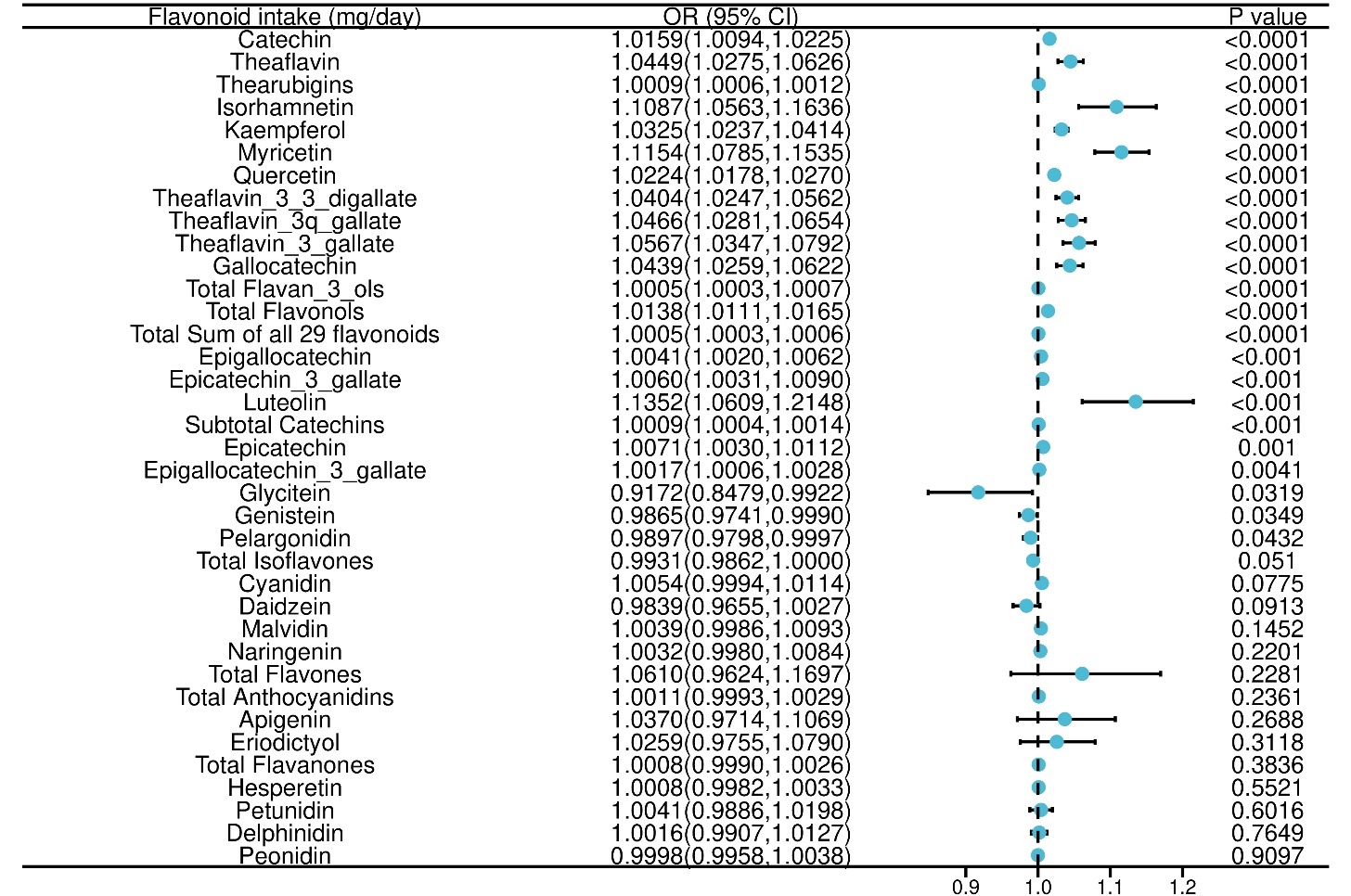


Supplementary Figure 1. Associations between flavonoid intake and risk of prediabetes by unadjusted Logistic regression.


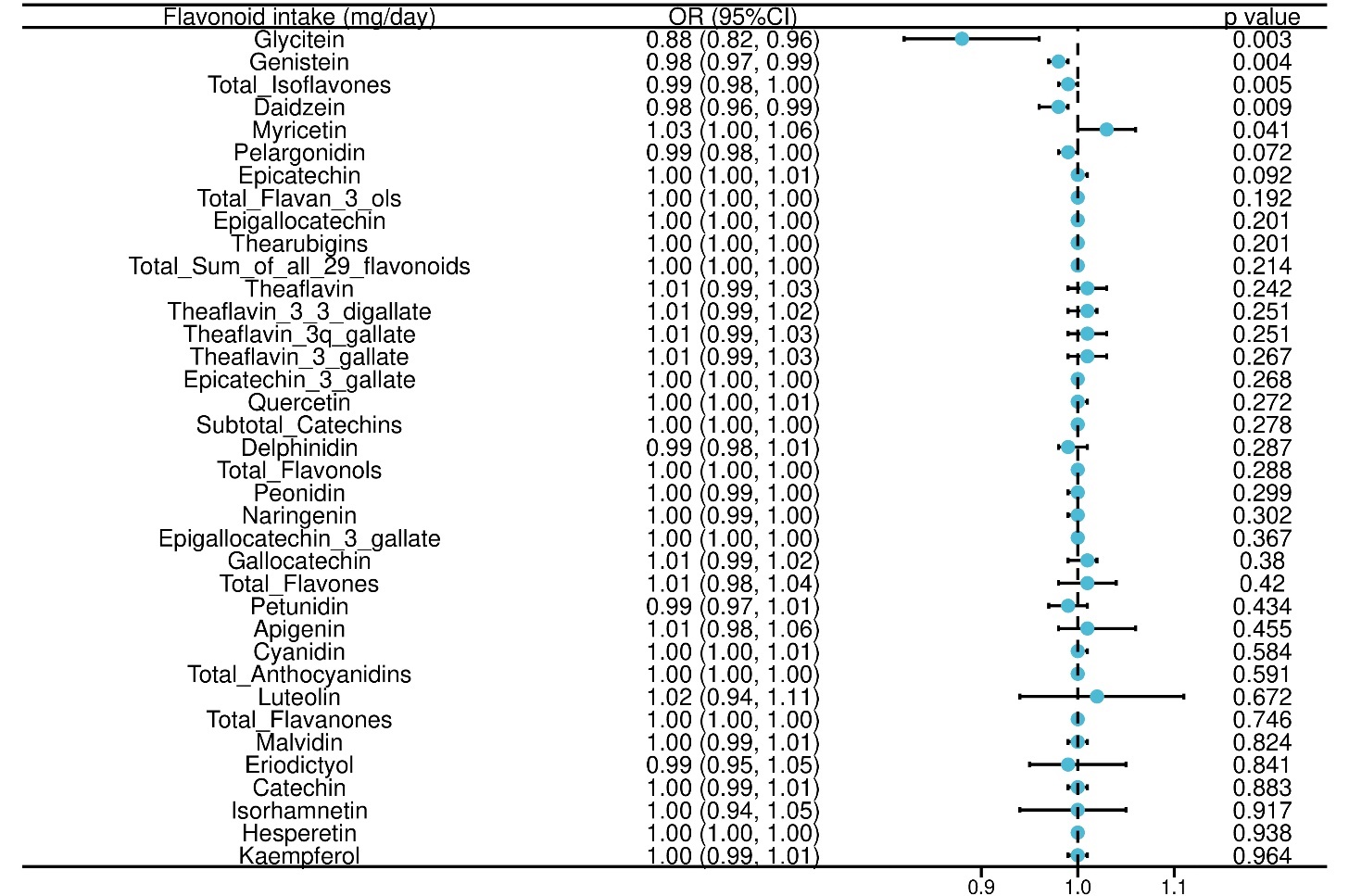


Supplementary Figure 2. Association between dietary flavonoid intake and prediabetes risk with adjustment for age, sex, race, BMI, daily energy intake, total time of PA, smoking habits, alcohol consumption, hyperlipidaemia, and hypertension. BMI body mass index, PA physical activity


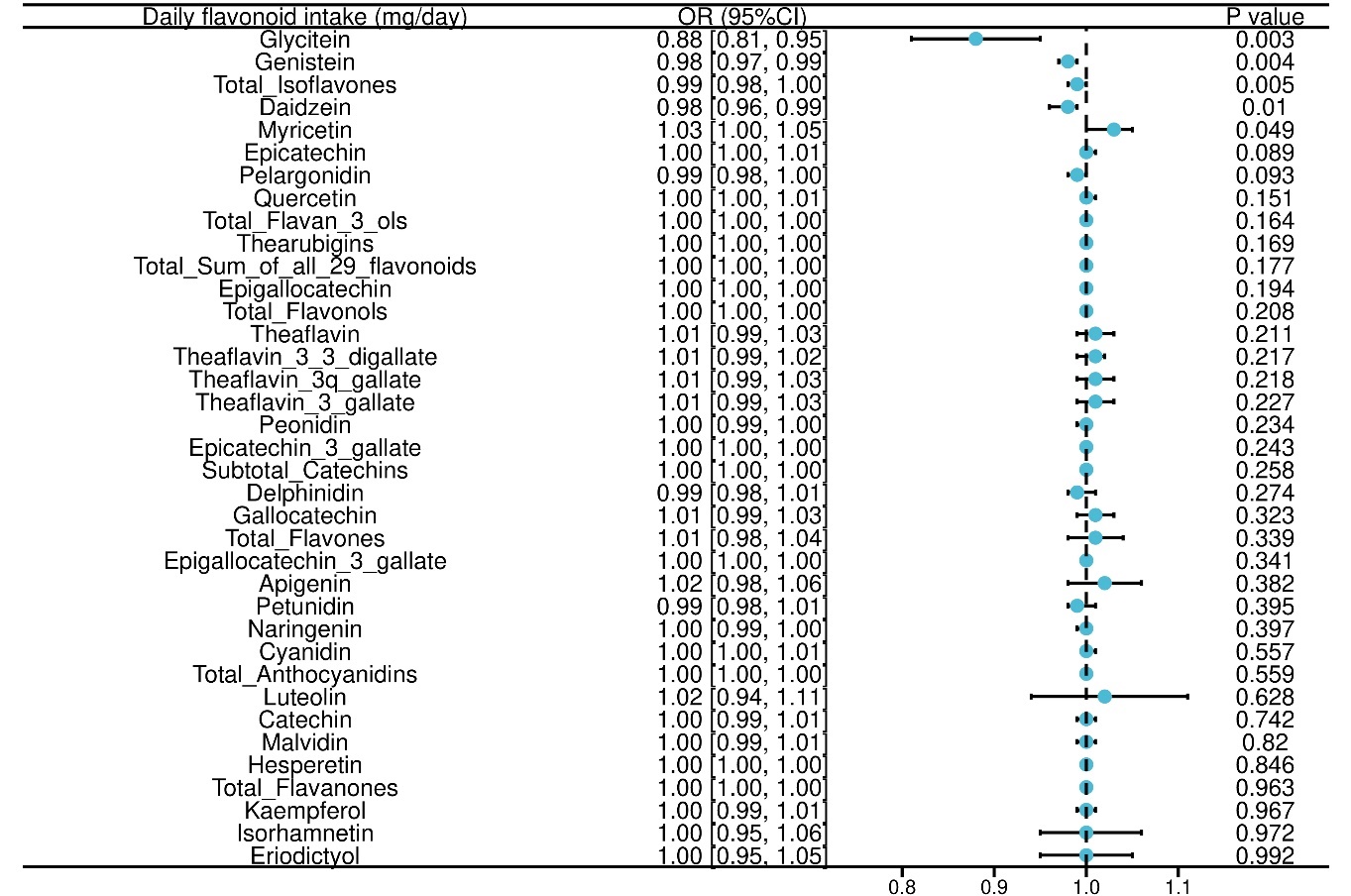


Supplementary Figure 3. Association between dietary flavonoid intake and prediabetes risk with adjustment for age, sex, race, BMI, daily energy intake, total time of PA, smoking habits, alcohol consumption, hyperlipidaemia, hypertension, and survey year cycle. BMI body mass index, PA physical activity


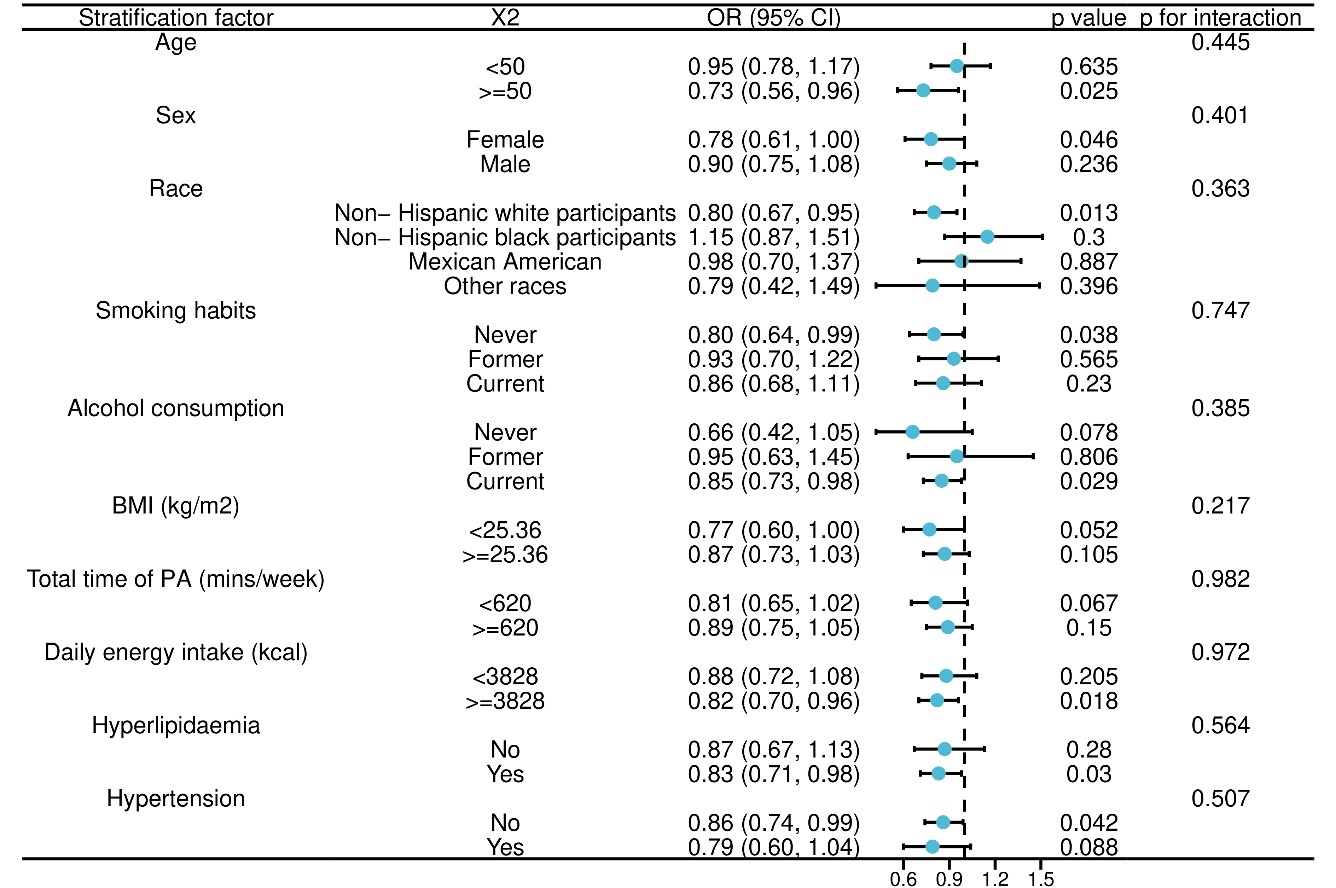


Supplementary Figure 4. Stratified analysis on the association between concentration of daidzein in urine and the risk of prediabetes with adjustment for age, sex, race, BMI, daily energy intake, total time of PA, smoking habits, alcohol consumption, hyperlipidaemia, hypertension, and creatinine in urine. The concentrations of urinary isoflavone metabolites were transformed by taking the logarithm (base 10). BMI body mass index, PA physical activity


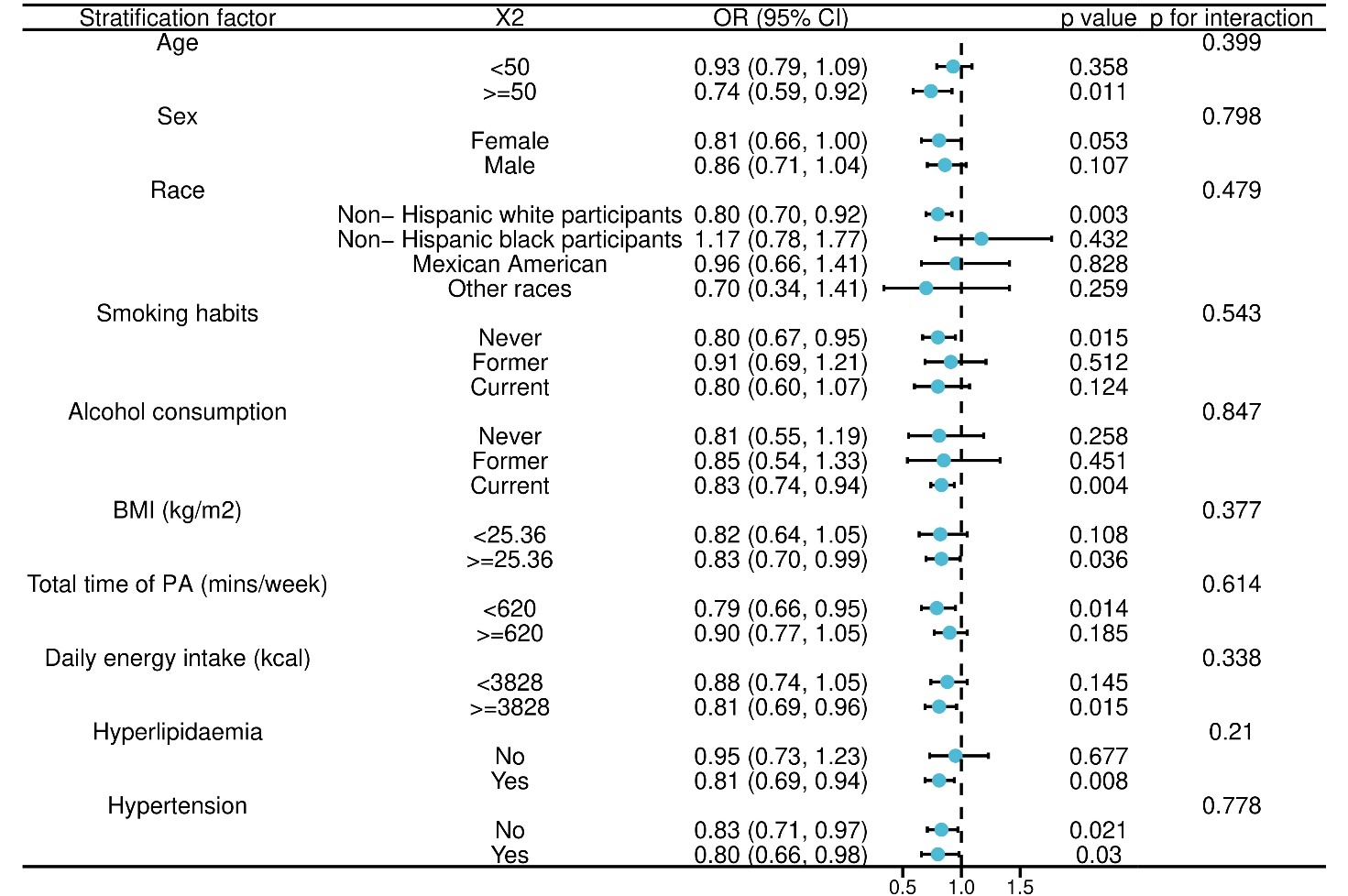


Supplementary Figure 5. Stratified analysis on the association between concentration of genistein in urine and the risk of prediabetes with adjustment for age, sex, race, BMI, daily energy intake, total time of PA, smoking habits, alcohol consumption, hyperlipidaemia, hypertension, and creatinine in urine. The concentrations of urinary isoflavone metabolites were transformed by taking the logarithm (base 10). BMI body mass index, PA physical activity
